# Indoxyl Sulfate Contributes to Progression of Renal Injury in the Postpartum Period Following Pregnancy-Related Acute Kidney Injury †

**DOI:** 10.3390/toxins18080350

**Published:** 2026-08-17

**Authors:** Ashley Griffin, Brittany Berry, Lidia Melaku, Delijah Johnson, Perla Guevarra, Leslie A. Shack, Shauna-Kay Spencer, Bindu Nanduri, Kedra Wallace

**Affiliations:** 1 Department of Pharmacology & Toxicology, University of Mississippi Medical Center, Jackson, MS 39216, USA; agriffin4@umc.edu (A.G.);; 2 Department of Obstetrics & Gynecology, University of Mississippi Medical Center, Jackson, MS 39216, USA; 3 Department of Comparative Biomedical Sciences, Mass Spectrometry Core Facility, Mississippi State University, Mississippi State, MS 39762, USA; shack@cvm.msstate.edu (L.A.S.); bnanduri@cvm.msstate.edu (B.N.)

**Keywords:** AKI, CKD, indoxyl sulfate, postpartum, pregnancy, renal injury, uremic toxin

## Abstract

Pregnancy-related acute kidney injury (PR-AKI) increases the risk of chronic kidney disease (CKD) in the postpartum period, yet mechanisms driving this transition remain unclear. Uremic toxins, including indoxyl sulfate (IS), are implicated in AKI-to-CKD progression. Using a rat model of PR-AKI induced by ischemia–reperfusion on gestational day (GD) 18, we assessed IS contributions to renal injury in the postpartum. A subset of rats received the oral adsorbent AST-120 in the postpartum period to reduce IS. Additional groups received IS during pregnancy with or without AST-120 treatment in the postpartum period. Renal function, blood pressure, circulating and urinary IS concentrations, and renal histopathology were evaluated. PR-AKI resulted in sustained postpartum elevations in circulating (*p* = 0.03) and urinary (*p* < 0.03) IS, reduced urine output (*p* = 0.03), increased proteinuria (*p* < 0.0001), increased serum albumin, and increased renal fibrosis (*p* = 0.008) compared to normal pregnant control rats. Absorption of indole, a precursor for IS, significantly reduced urinary IS (*p* = 0.03), reduced serum creatinine (*p* = 0.006), and attenuated renal fibrosis (*p* = 0.002) in treated PR-AKI rats. While not significant, indole absorption improved urine output (*p* = 0.06) and reduced proteinuria (*p* = 0.07) in treated PR-AKI rats. IS administration during pregnancy recapitulated key features of postpartum CKD. Elevated IS contributes to persistent renal injury following PR-AKI. Postpartum reduction in IS with AST-120 dampens the progression of renal injury. These findings highlight uremic toxins as mechanistic drivers and potential therapeutic targets in post partum CKD following PR-AKI.

## 1. Introduction

Acute kidney injury occurring during pregnancy (PR-AKI) impacts up to 2% of pregnancies in developing countries and is associated with maternal and perinatal morbidity and mortality, such as hypertensive disorders of pregnancy and fetal growth restriction [1,2,3]. Women with a history of PR-AKI have an increased risk of long-term renal injury (52-fold) and development of cardiovascular disease (23-fold) when assessed up to two years postpartum [4]. PR-AKI increases the risk of developing chronic kidney disease (CKD), adverse cardiovascular events (i.e., myocardial infarction and stroke), and cognitive decline [5,6]. Despite the increased risk of adverse events for women with a history of PR-AKI, the pathophysiologic mechanisms and potential therapeutic interventions on the progression of renal injury following PR-AKI remain understudied.

Uremic toxins are metabolic waste products that accumulate in the body following kidney damage. It is well documented that uremic toxins are involved in the progression from AKI to CKD [7,8,9,10]. Accumulation of uremic toxins following kidney injury is a marker of impaired renal clearance and actively contributes to the progression of renal disease [10]. In non-pregnant populations, it is well documented that uremic toxins contribute to renal dysfunction by activating profibrotic pathways, inducing oxidative stress and proinflammatory pathways, downregulating anti-aging proteins, and causing mitochondrial dysfunction [11,12,13]. These processes lead to renal fibrosis, cellular senescence, changes in blood pressure, and dysfunction in kidney clearance [11,12,13]. Indoxyl sulfate (IS) is a protein-bound uremic toxin associated with declining renal function and neurological complications associated with CKD [7,10]. Whether IS accumulation resulting from PR-AKI contributes to renal injury progression during the postpartum period has not been established. Given the increasing incidence of renal injury during pregnancy (e.g., preeclampsia) and its association with the development of CKD among this population of postpartum women [14], this remains a gap in knowledge.

Our animal model of PR-AKI uses renal ischemia/reperfusion to induce AKI during pregnancy, which results in increased renal fibrosis, decreased urine output, and elevated blood urea nitrogen and creatinine levels [15]. Additionally, postpartum studies from PR-AKI animals from our lab have confirmed increased renal fibrosis, urine creatinine, and proteinuria [16]. Given the established role of IS in the progression of non-pregnancy-related CKD, interventions aimed at decreasing circulating levels of IS may be a promising strategy to test in the PR-AKI population and among women with renal-related pregnancy disorders. AST-120 is an oral carbon charcoal adsorbent that decreases levels of IS via adsorption of its precursor, indole. When AST-120 was administered orally, non-pregnant patients with CKD showed a slower decline in renal function and a decrease in both circulating uremic toxins and creatinine levels [17]. AST-120 has also been shown to attenuate renal injury and improve kidney function in non-pregnant animal models of CKD by reducing IS [18].

While these studies are promising and suggest that targeting IS may be a viable therapeutic strategy, whether this extends to postpartum kidney health following PR-AKI is unknown. To address this gap, we tested the hypothesis that IS accumulation is responsible for progressive renal injury in the postpartum period following PR-AKI and that preventing IS accumulation attenuates renal injury. We evaluated this hypothesis via two approaches: (1) by administering AST-120 to rats with a history of PR-AKI and (2) through direct IS treatment to normal pregnant rats to determine if pharmacological administration of IS can replicate renal injury similar to what is seen among AKI rats.

## 2. Results

### 2.1. Impact of AST-120 Treatment in PR-AKI Rats

#### 2.1.1. PR-AKI Does Not Significantly Impair Litter Outcomes but Results in Increased Postpartum Blood Pressure

**Figure 1:**
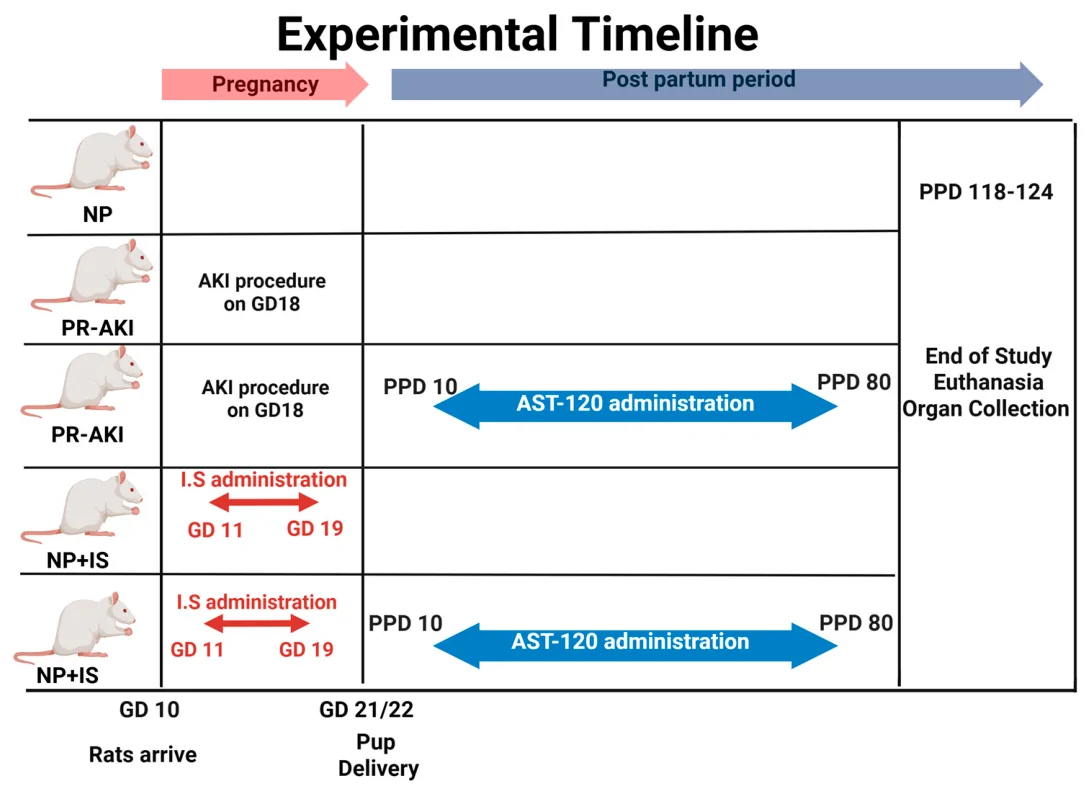
Experimental timeline. Rats arrived on gestational day (GD)10. PR-AKI rats receive the AKI procedure on GD18. IS-treated rats receive IS from GD11 until GD19. Rats delivered between GD21-22. AST-120 administration began on postpartum day (PPD) 10 and ended on 80. The end-of-study measurements were performed between PPD 118-124. The *N*’s for the groups are the following: NP (27), PR-AKI (20), PR-AKI+AST-120 (14), NP+IS (15), and NP+IS+AST-120 (9). Created in BioRender. Melaku, L. (2026) https://BioRender.com/27oi62o.

Figure 1 presents the timeline of procedures in this study. Compared to normal pregnant (NP; control group) rats, PR-AKI did not significantly reduce pup birth weight (6.91 ± 0.07 g vs. 6.72 ± 0.10 g; *p* = 0.11) or the litter size (10.95 ± 0.61 vs. 10.00 ± 0.66; *p* = 0.30). There was no significant difference in litter survival rates between PR-AKI (93.9%) and NP litters (99.3%; *p* = 0.06). Similarly, to what we previously reported, PR-AKI is associated with early postpartum mortality, with 30.1% of rats dying before postpartum week 2 vs. NP rats (*p* = 0.001).

AST-120 treatment did not begin until the postpartum period; therefore, all PR-AKI rats’ baseline data are reported together at GD19. Body weight was measured throughout the study, and time effect (F_1.724,32.75_ = 11.7; *p* = 0.0003) was significant, but no group (F_2,87_ = 2.52; *p* = 0.09) effect or interaction effect (F_3.45,32.75_ = 0.73; *p* = 0.56) was significant. Post hoc analysis indicated that only the GD19 time point showed significant differences between groups. PR-AKI rats (288.5 ± 4.65 g) weighed significantly less than NP rats (307.2 ± 6.09 g; *p* = 0.02) at GD19. Mean arterial pressure (MAP) was measured throughout the study, and time (F_1.93,139.1_ = 12.23; *p* < 0.0001) and group (F_2,144_ = 6.41; *p* = 0.002) effects were significant, while the interaction effect (F_3.86,139.1_ = 1.04; *p* = 0.39) was not significant. When measuring MAP, PR-AKI rats had increased MAP at postpartum month (PPM) 4 compared to NP rats (*p* = 0.05, Table 1). AST-120 treatment in PR-AKI rats decreased MAP compared to untreated PR-AKI rats at PPM2 (*p* = 0.01; Table 1). However, by PPM4 (5 weeks following cessation of AST-120 treatment), this significant difference between NP and PR-AKI no longer existed (*p* = 0.06).

#### 2.1.2. Indoxyl Sulfate Is Increased Following PR-AKI

**Figure 2:**
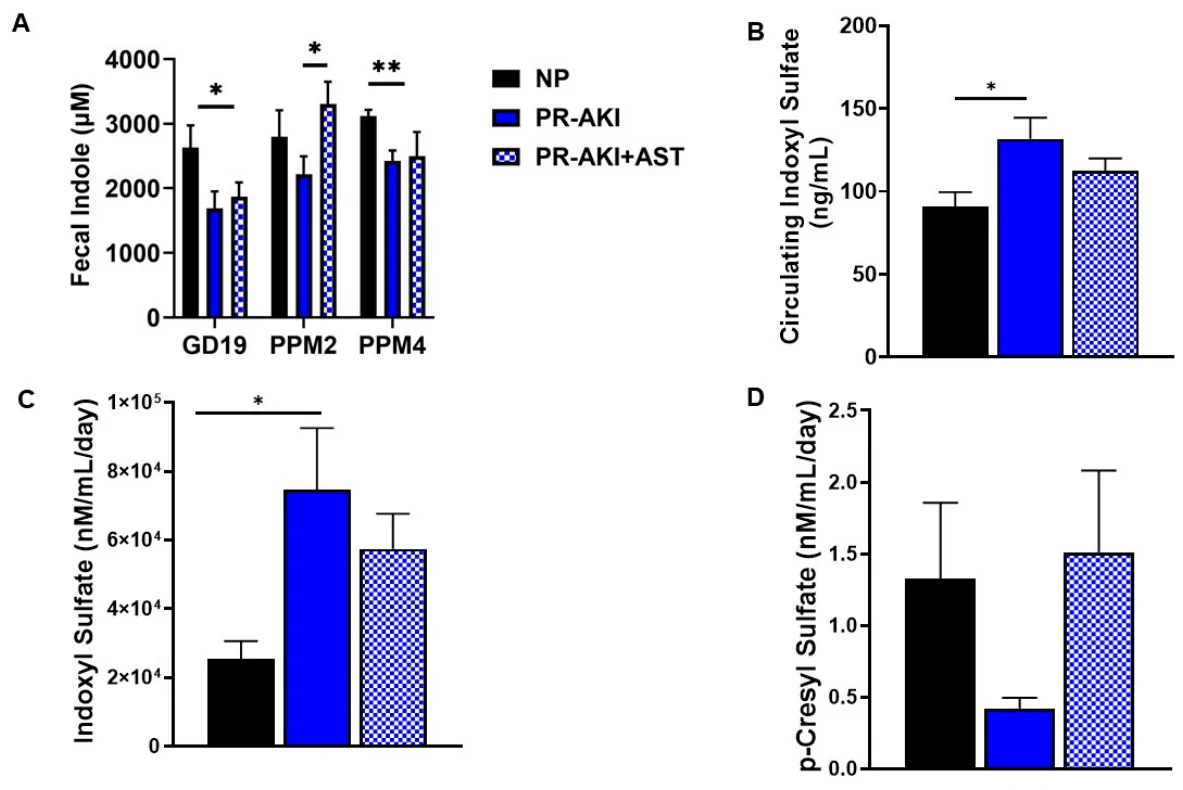
Indole and uremic toxin concentrations in PR-AKI rats. (**A**) AST-120 restores fecal indole concentrations compared to PR-AKI rats at PPM2 and PPM4. (**B**) Total circulating IS levels are higher in PR-AKI rats compared to NP rats at PPM4. (**C**) PR-AKI rats excreted higher urinary IS concentrations at PPM4 than NP rats. (**D**) There was no significant difference in the concentration of pCS excreted at PPM4 between groups. LC-MS data were normalized to urine output and is represented as (nM of toxin/mL/day). * *p* < 0.05, ** *p* < 0.005. GD = Gestational day, PPM = postpartum month. Samples from *n* = 8–9 per group were used to determine indole levels via assay, *n* = 5–8 per group were used for circulating IS via ELISA, and *n* = 9–10 per group for urinary IS and PCS levels via LC-MS.

We measured indole an IS precursor levels in fecal samples. Overall, there was a significant time (F_1.79,37.59_ = 6.09; *p* = 0.007) and group effect (F_2,23_ = 3.89; *p* = 0.04), but no significant interaction effect (F_3.58,3759_ = 1.81; *p* = 0.15). Throughout the study, PR-AKI rats had decreased fecal indole excretion (*p* = 0.05; Figure 2A). Administration of AST-120 was restored at PPM2 (*p* = 0.02 vs. PR-AKI); however, by PPM4, fecal indole levels in this group had decreased to levels comparable to those in untreated PR-AKI rats (*p* = 0.85 vs. PR-AKI; Figure 2A).

To determine whether systemic IS levels increased during the postpartum period and whether AST-120 prevented any increase, circulating IS was measured. PR-AKI rats had significantly more circulating IS than NP rats (*p* = 0.02; Figure 2B). When measured 5 weeks post-AST-120, cessation did not significantly decrease IS in response to renal injury. To determine if IS was being cleared from the body, urinary IS excretion was also measured. There was a significant difference with PR-AKI rats and PR-AKI+AST-120 rats excreting more urinary IS than NP rats (*p* < 0.03 and *p* < 0.01, respectively; Figure 2C). *p*-Cresol sulfate (pCS) is another uremic toxin that has been implicated in the transition from AKI-to-CKD, and can be decreased by AST-120 treatment [13,19]. We also measured *p*-Cresol sulfate (pCS) levels, another prevalent uremic toxin in CKD, in urine and found no significant difference among the groups (*p* = 0.20; Figure 2D). An important finding of the current study is that PR-AKI rats excreted significantly more IS than pCS (74,580 ± 18,019 nM/mL/day vs. 0.42 ± 0.07 nM/mL/day; *p* < 0.003).

#### 2.1.3. Elevated Indoxyl Sulfate Is Associated with Progressive Renal Injury in the Postpartum Period

We measured urine output, an indicator of renal function, throughout the study and found a group effect (F_2,76_ = 8.62; *p* = 0.0004), but no time effect (F_1.88,62.95_ = 2.91; *p* = 0.07) or interaction effect (F_3.76,62.95_ = 1.80; *p* = 0.14). Since there was no time effect, we assessed each time point independently. PR-AKI rats had significantly lower urine output than NP rats throughout the study (*p* < 0.05; Table 1). AST-120 restored urine output versus untreated PR-AKI animals, but this was not significant at PPM4 (*p* = 0.06; Table 1). There was a significant group effect (F_2,81_ = 17.37; *p* < 0.0001), but no time effect (F_1.47,59.55_ = 2.17; *p* = 0.14) or interaction effect (F_2.94,59.55_ = 0.64; *p* = 0.59) of proteinuria. Throughout the study, PR-AKI rats had significantly higher proteinuria than NP rats (*p* = 0.001; Table 1). At PPM2, AST-120 treatment prevents the increase in proteinuria compared with untreated PR-AKI rats, but it was not statistically significant (*p* = 0.07; Table 1). We measured the urinary protein/creatinine ratio throughout the study, and there was a significant group effect (F_2,30_ = 5.14; *p* = 0.01), but no time (F_1.38,20.70_ = 2.78; *p* = 0.10) or interaction effect (F_2.76,20.70_ = 0.62; *p* = 0.60). PR-AKI rats have a higher protein/creatinine ratio than NP rats at GD19 (*p* = 0.02; Table 1) and PPM2 (*p* = 0.007; Table 1). At PPM2, AST-120 treatment prevents increases in the urinary protein/creatinine ratio in PR-AKI rats (*p* = 0.02; Table 1). At PPM4, there are no significant differences between groups.

**Figure 3:**
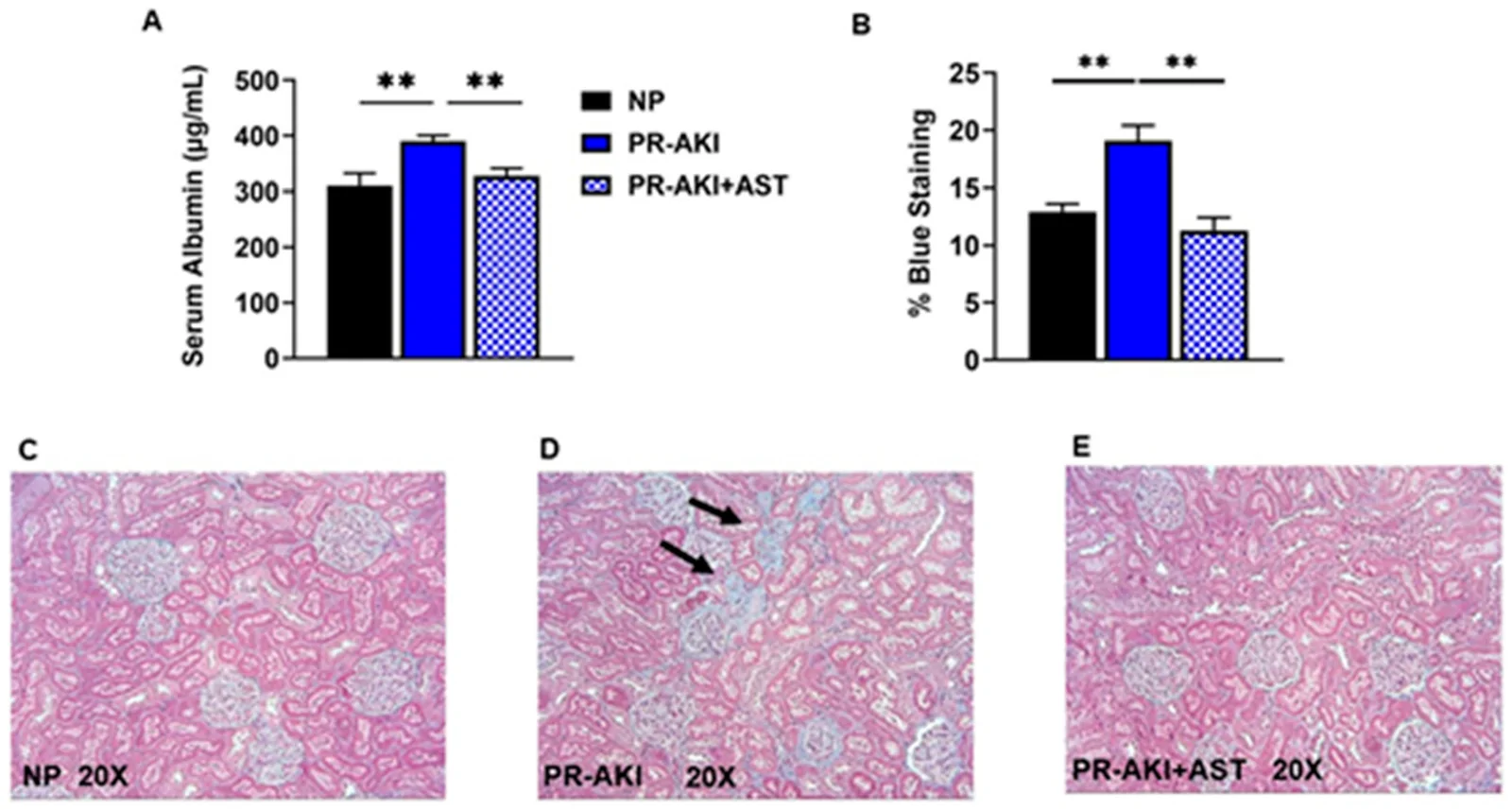
Renal outcomes. (**A**) Serum albumin is increased in PR-AKI rats compared to NP and PR-AKI+AST-120 rats. (**B**) PR-AKI rats showed greater fibrosis than NP and PR-AKI+AST-120 rats. Representative images (**C**) NP, (**D**) PR-AKI, (**E**) PR-AKI+AST-120 ** *p* < 0.005 GD = Gestational day, PPM = postpartum month. Samples from *n* = 6–12 per group for serum albumin and *n* = 4 per group with 10 images per kidney for renal fibrosis. Black arrows are used to point out areas of increased blue staining.

Serum creatinine was increased in PR-AKI rats compared to NP (*p* = 0.008) and PR-AKI+AST-120 (*p* = 0.006; Table 1) rats at PPM4. AST-120 rats’ serum creatinine levels were restored to levels comparable to NP (*p* = 0.55). PR-AKI rats had a significantly increased kidney weight (0.41 ± 0.02 g) compared to NP (*p* = 0.01; 0.33 ± 0.01 g), which AST-120 treatment did not prevent (*p* = 0.72; 0.39 ± 0.02 g). Serum albumin was increased in PR-AKI rats compared to NP (*p* = 0.007) and PR-AKI+AST-120 (*p* = 0.004; Figure 3A) rats. Regarding renal fibrosis, we found a significant difference between groups (*p* = 0.0004). PR-AKI rats showed greater renal fibrosis than NP rats (*p* = 0.001; Figure 3B) and AST-120 (*p* = 0.002; Figure 3B). Representative images of the renal fibrosis staining are shown in Figure 3C–E. These renal outcomes together indicate that postpartum AST-120 treatment can decrease renal injury.

### 2.2. Impact of AST-120 Treatment in IS-Treated Rats

#### 2.2.1. IS Administration Does Not Significantly Impair Litter Outcomes or Increase MAP

Compared to NP rats, NP+IS did not significantly reduce pup birth weight (6.91 ± 0.07 g vs. 6.82 ± 0.09 g; *p* = 0.39) or the litter size (10.95 ± 0.61 vs. 12.44 ± 0.72; *p* = 0.12). There was no significant difference in litter survival rates between NP+IS (91.5%) and NP litters (99.3%; *p* = 0.18). IS administration during pregnancy was not associated with increased mortality vs. NP rats with only 0.7% mortality (*p* = 0.16). Body weight was measured throughout the study, and the time effect (F_1.65,37.19_ = 25.15; *p* < 0.0001) was significant, but the group (F_2,50_ = 1.90; *p* = 0.16) and the interaction (F_3.31,37.19_ = 0.98; *p* = 0.41) effect were not. When measuring MAP at PPM2, AST-120 treatment in NP+IS rats restored MAP compared to untreated NP+IS rats (*p* = 0.04; Table 2) at PPM2. However, by PPM4, AST-120 treatment no longer significantly prevents the increase in MAP in NP+IS rats following AST-120 cessation (*p* = 0.05; Table 2).

#### 2.2.2. AST-120 Attenuates Renal Dysfunction in the Postpartum Period Among IS-Administered Pregnant Rats

**Figure 4:**
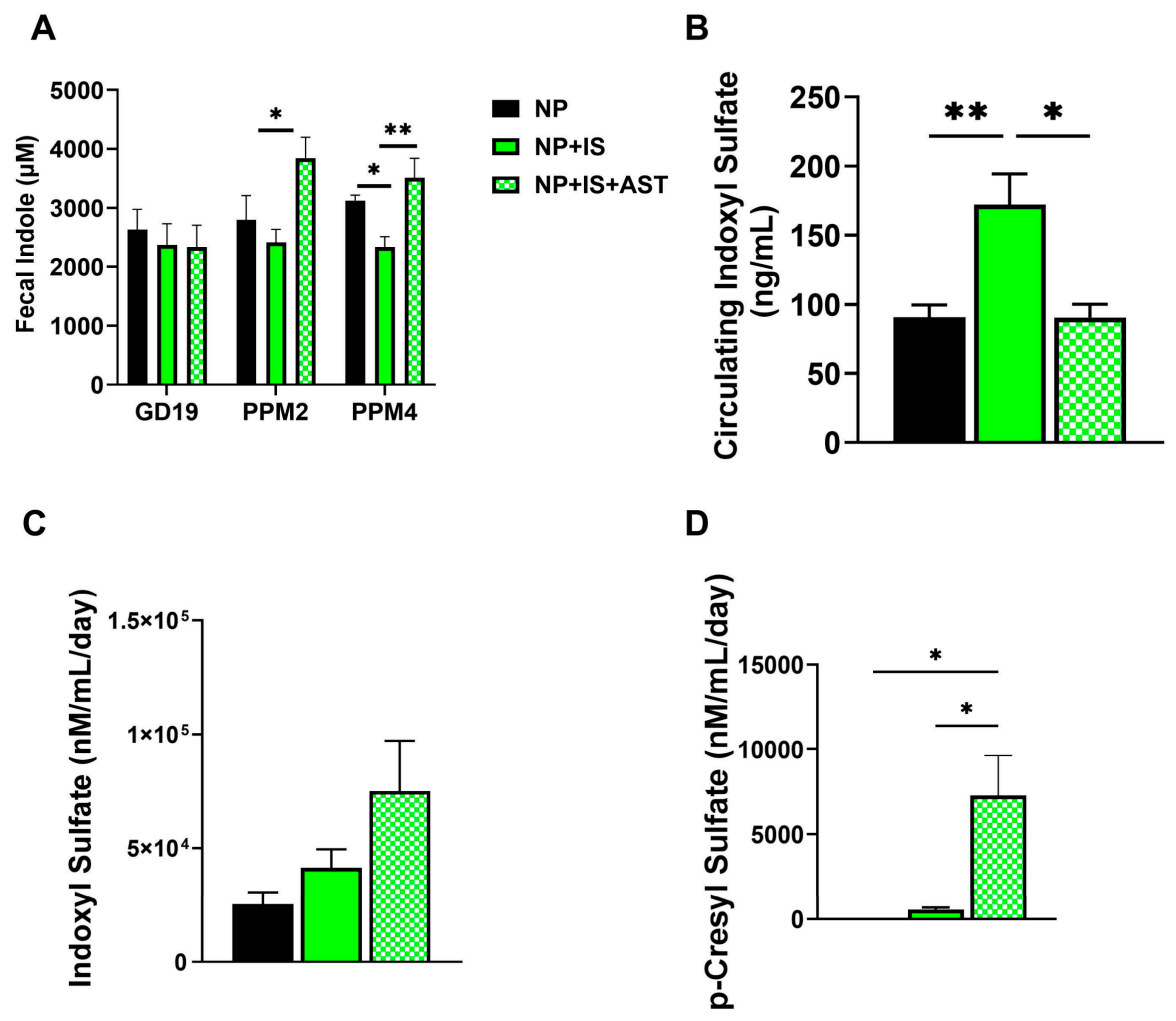
Indole and uremic toxin concentrations in NP+IS rats. (**A**) AST-120 increases fecal indole concentrations compared to NP+IS rats at PPM2 and PPM4. (**B**) Total circulating IS levels are higher in NP+IS rats compared to NP and AST-120 rats at PPM4. (**C**) Urinary IS concentrations were not significantly different between groups at PPM4. (**D**) NP+IS+AST-120 rats excreted more pCS than NP+IS and NP rats. LC-MS data were normalized to urine output and is represented as (nM of toxin/mL/day). * *p* < 0.05, ** *p* < 0.005. GD = Gestational day, PPM = postpartum month. Samples from *n* = 6–8 per group were used to determine indole levels via assay, *n* = 4–7 per group were used for circulating IS via ELISA, and *n* = 10 per group for urinary IS and pCS levels via LC-MS.

To further investigate the role of increased IS during pregnancy on renal function, we also administered IS to a cohort of pregnant rats. We have previously reported that IS administration during pregnancy leads to a renal injury phenotype similar to the PR-AKI model. When measuring fecal indole, there was a significant group effect (F_2,21_ = 4.91; *p* = 0.02), but no significant time effect (F_1.96,32.36_ = 3.14; *p* = 0.06) or interaction effect (F_3.92.32.36_ = 1.77; *p* = 0.16). By PPM4, NP+IS rats had significantly decreased indole vs. NP rats; however, NP+IS+AST-120-treated rats had significantly increased indole levels vs. untreated NP+IS rats both during (*p* = 0.01) and after treatment (*p* = 0.004; Figure 4A). There was a significant difference in circulating IS; AST-120 treatment following IS administration during pregnancy restores circulating IS at PPM4 compared with non-treated NP+IS rats (*p* = 0.03; Figure 4B). When measuring urinary IS, there was a significant difference between groups (*p* < 0.0001; Figure 4C). There was a significant difference in urinary pCS between groups (*p* = 0.008). NP+IS+AST-120 rats excreted significantly higher urinary pCS than NP (*p* < 0.01; Figure 4D) and NP+IS rats (*p* = 0.03; Figure 4D). Rats that were administered IS excreted significantly more IS than pCS (*p* = 0.003).

**Figure 5:**
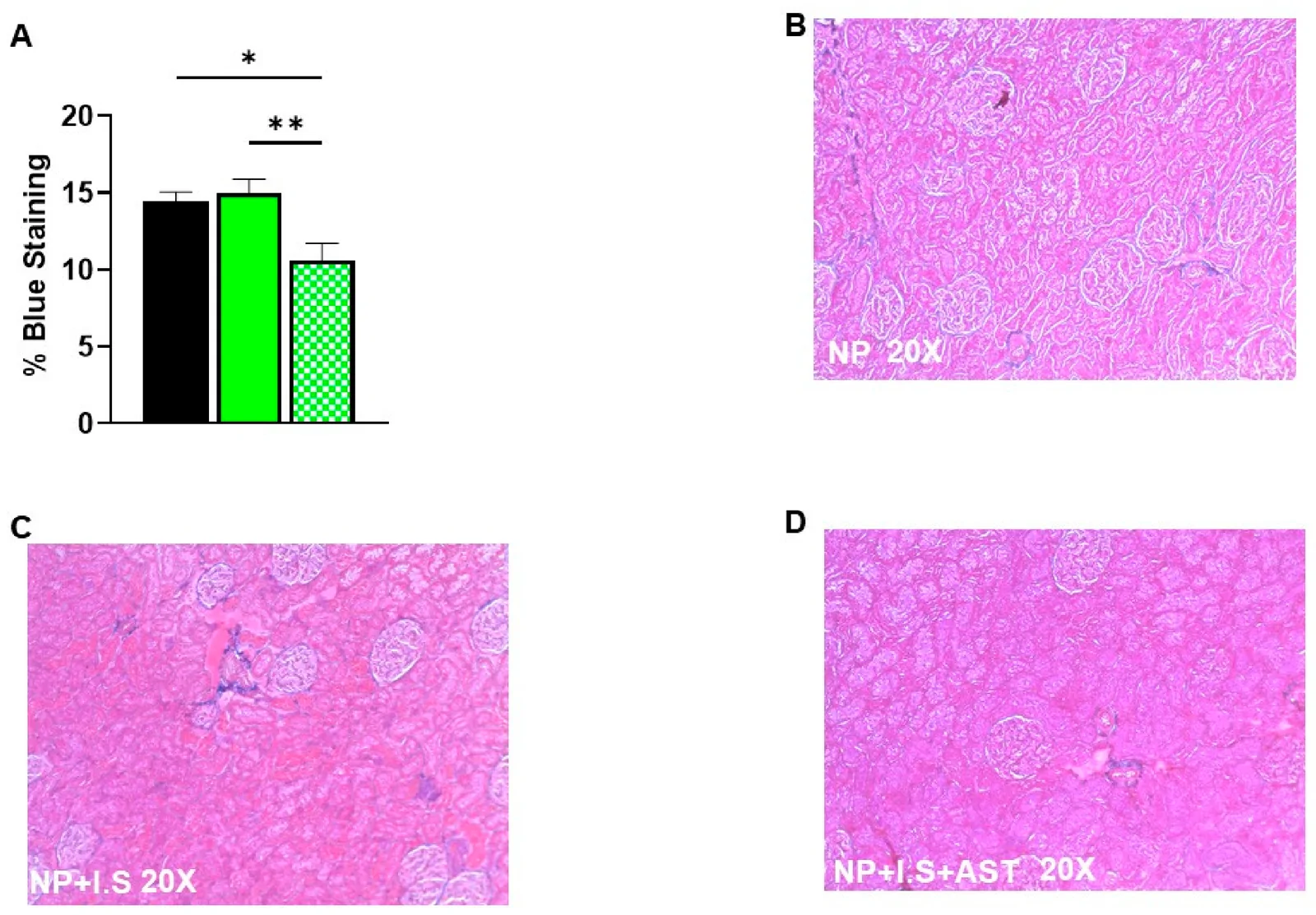
Renal outcomes. (**A**) AST-120 treatment decreased renal fibrosis in NP+IS rats. Representative images (**B**) NP, (**C**) NP+IS, (**D**) NP+IS+AST-120 * *p* < 0.05, ** *p* < 0.005. GD = Gestational day, PPM = postpartum month. Samples from *n* = 3–4 per group with 10 images per kidney for renal fibrosis. The experimental groups in Figure 5A are identical to those shown in Figure 4; group designations and corresponding colors are listed in Figure 4A.

To further investigate the role of increased IS during pregnancy on postpartum renal function, we also administered IS to a cohort of pregnant rats. We have previously shown that IS administration during pregnancy leads to a renal injury phenotype similar to the PR-AKI model. We measured urine output, an indicator of renal function, throughout the study (Table 2) and found a group effect (F_2,114_ = 10.34; *p* < 0.0001), but no time effect (F_2,114_ = 2.061; *p* = 0.13) or interaction effect (F_4,114_ = 0.61; *p* = 0.65). Since there was no time effect, we assessed each time point independently. At GD19, NP+IS had significantly lower urine output than NP rats (*p* < 0.0001; Table 2). At PPM2, there was a significant difference in urine output between groups (*p* = 0.007). Compared to NP rats, NP+IS (*p* = 0.01) and NP+IS+AST-120 rats (*p* = 0.03; Table 2) had a significantly lower urine output. At PPM4, there was no difference in urine output between groups. We measured proteinuria, another indicator of renal function, throughout the study (Table 2) and found a group effect (F_2,82_ = 20.28; *p* < 0.0001), but no time effect (F_2,82_ = 1.51; *p* = 0.23) or interaction effect (F_4,82_ = 2.31; *p* = 0.06). Thus, we assessed each time point independently. Untreated NP+IS rats had higher proteinuria than NP rats at GD19 (*p* = 0.002), PPM2 (*p* = 0.003), and PPM4 (*p* = 0.0007; Table 2). At PPM2, AST-120-treated NP+IS rats still had significantly higher proteinuria compared with NP rats (*p* < 0.0001; Table 2). At PPM4, AST-120 treatment prevents the increase in proteinuria compared to untreated IS rats (*p* = 0.03; Table 2). We also measured the urinary protein/creatinine ratio, and there was no significant group (F_2,51_ = 1.5; *p* = 0.23), time (F_1.67,42.54_ = 0.40; *p* = 0.64), or interaction effect (F_3.34,42.54_ = 0.22; *p* = 0.90). Serum creatinine was increased in NP+IS rats (*p* = 0.01; Table 2). AST-120 treatment decreased serum creatinine compared to untreated NP+IS rats (*p* = 0.008; Table 2). Regarding renal fibrosis, there was a significant difference between groups (*p* = 0.004). AST-120 decreased compared to NP (*p* = 0.01; Figure 5A) and NP+IS (*p* = 0.005; Figure 5B). Representative images are presented in Figure 5B–D. IS did not lead to renal fibrosis compared to NP rats.

## 3. Discussion

PR-AKI is increasingly recognized as an essential contributor to long-term maternal kidney disease, yet the biological mechanisms underlying postpartum disease progression remain poorly defined [2]. This study tested the hypothesis that elevated IS contributes to the persistence of renal injury in the postpartum period following PR-AKI. The findings from this study indicated that IS contributes to the progression of renal injury following PR-AKI. Together, these findings support a mechanistic role for uremic toxins, specifically IS, in driving the AKI-to-CKD transition after pregnancy.

Consistent with prior clinical and experimental studies linking AKI to later CKD, rats with a history of PR-AKI in the present study exhibited reductions in urine output, increased proteinuria and serum creatinine, and enhanced renal fibrosis months after delivery [16]. Uremic toxins have emerged as key mediators of maladaptive repair following AKI, promoting oxidative stress, inflammation, endothelial dysfunction, and fibrotic remodeling [20]. Our findings demonstrated that PR-AKI results in sustained postpartum accumulation in IS, a protein-bound uremic toxin strongly associated with renal disease severity and adverse outcomes in both AKI and CKD, and AST-120 minimizes the progression of renal injury by reducing IS levels.

IS originates from gut-derived indole and accumulates when renal clearance is impaired [21]. In the current study, PR-AKI animals exhibited reduced fecal indole levels during late gestation and postpartum, coupled with increased urinary and circulating IS, suggesting altered gut–kidney handling of tryptophan metabolites following renal ischemic injury in pregnancy. Upregulation of the renal organic anion transporters (OAT) 1 and 3 may occur as a compensatory response to elevated circulating IS [22]. Mediating basolateral uptake into proximal tubules leads to increased urinary IS excretion even while circulating concentrations remain elevated [22]. Thus, urinary IS reflects both systemic burden and tubular secretory capacity. Significantly, postpartum treatment with AST-120 reduced the precursor indole, decreased urinary IS, and normalized renal outcomes, supporting a causal role for IS in persistent renal injury. These findings align with prior studies demonstrating that AST-120 attenuates proteinuria, uremic toxin concentrations, and tubulointerstitial injury in non-pregnant models of renal disease, but our study extends these observations to a postpartum, female-specific context that has been historically underrepresented in preclinical nephrology research [23]. The reduction in urinary IS has been seen in a unilateral nephrectomy model of renal injury 2 weeks after the initiation of AST-120 treatment supporting the reduction in urinary IS seen in our AST-120 treated animals [24]. AST-120 treatment decreased circulating IS, even after the cessation of treatment, compared to untreated PR-AKI rats, which could be different from clinical data due to the postpartum period being a vulnerable period. A recent study in patients with end-stage renal disease undergoing hemodialysis found that premature AST-120 cessation leads to a rebound increase in circulating IS [25]. Given that our animal model focuses on the postpartum period, more mechanistic studies will be needed to determine whether our model will exhibit the rebound effect reported after the cessation of AST-120.

Levels of urinary pCS were significantly lower than IS levels in PR-AKI rats, confirming studies from non-pregnant populations indicating that IS plays a primary role in renal injury while pCS has been found to be more associated with the cardiovascular events following CKD. [8]. The selected dose (5 g/100 g chow) of AST-120 was based on prior studies conducted in virgin female rats [26,27], as these provide the most established framework for IS modulation. However, the physiological changes associated with pregnancy and the postpartum period may influence pharmacokinetics, and this dose may not represent the optimal regimen for the postpartum model. It should be noted that AST-120 reduces circulating IS; it also affects other molecules, so its benefits cannot be attributed solely to IS [28,29]. Other uremic toxins may have assisted in the effects we saw in our study, so measuring a range of toxins would strengthen the conclusions of the study.

A potential limitation of this study is that circulating levels of IS were measured by ELISA, rather than by the gold-standard mass spectrometry method. In addition, we did not evaluate circulating pCS levels. To truly understand the impact of uremic toxins, IS and pCS on postpartum renal function, future studies will better evaluate these toxins and examine mechanisms inclusive of gut metabolism, transporter regulation and conversion of indole and p-cresol.

Pregnancy decreases serum albumin levels, particularly in the third trimester, whereas the early postpartum period is characterized by a rapid increase in albumin levels due to changes in fluid dynamics [30]. In addition, the bilateral renal ischemia–reperfusion model of AKI is a tubular renal injury model, which leads to low molecular weight proteins being leaked into the urine, as opposed to a glomerular renal injury model, which would lead to large molecular proteins, such as albumin, leaking into the urine [31,32]. IS has previously been implicated in the increasing of the production of albumin through hepatocytes, which is another possible mechanism to support the increase in serum albumin following PR-AKI [33]. Previously, this PR-AKI model showed no change in glomerular filtration rate at postpartum week 16 [16]. Serum creatinine was elevated in the PR-AKI and NP+IS models, indicating kidney dysfunction. AST-120 treatment decreased serum creatinine to levels comparable to those observed in NP rats, as previously observed [18,34]. In this study, AKI was associated with higher MAP than in NP animals, similar to our previous findings [15]. Although multiple regions per kidney were analyzed to account for tissue heterogeneity when measuring renal fibrosis, the relatively small number of biological replicates limits the strength of conclusions regarding fibrosis as a primary outcome. The ability of IS administration during pregnancy to induce postpartum renal dysfunction in otherwise normal pregnancies further strengthens the mechanistic link between uremic toxins and CKD progression. Previously, we reported that 8 days of IS administration during pregnancy led to decreased urine output and increased proteinuria [35]. In the postpartum period, NP animals treated with IS during pregnancy exhibited reductions in urine output, increased proteinuria and serum creatinine, and evidence of renal dysfunction comparable to PR-AKI animals, indicating that elevated IS is not merely a biomarker of renal damage but an active contributor to disease pathogenesis. Additionally, among NP+IS rats, the increased indoxyl sulfate production coincides with the decreased indole excretion, suggesting more indole is being absorbed within the gut epithelium [36]. Administration of AST-120 to NP+IS rats increased fecal indole levels and decreased circulating IS. The increase in fecal indole levels in NP+IS+AST-120 rats was expected as AST-120 adsorption binds free indole within the lumen, resulting in increased fecal excretion of indole [36]. Similar findings were reported by Sato et al., who reported increased fecal indole levels, but not increased fecal p-cresol levels in male mice with renal failure treated with AST-120 for four weeks [37]. Overall, these findings of renal dysfunction are consistent with experimental data that have previously demonstrated that IS promotes renal dysfunction by activating proinflammatory and profibrotic signaling pathways, including aryl hydrocarbon receptor-dependent mechanisms [38,39].

## 4. Conclusions

In summary, this study identifies IS as a key mediator of persistent renal injury following PR-AKI. Importantly, this is the first study to evaluate the therapeutic efficacy of postpartum AST-120 administration following PR-AKI, directly targeting uremic toxin accumulation during the critical postpartum recovery period. By demonstrating that postpartum reduction in IS improves renal outcomes and that gestational exposure to IS is sufficient to induce postpartum renal dysfunction, these findings provide an initial mechanistic insight into the AKI-to-CKD transition in pregnancy and the postpartum period. A future direction is to measure the downstream activations of proinflammatory and profibrotic signaling pathways, as well as the expressions of OAT1/3. IS has been implicated in blood–brain barrier disruption and neuroinflammation in experimental renal disease, raising the possibility that sustained postpartum elevations in uremic toxins contribute to neurological sequelae after PR-AKI [40]. Given the association between CKD and neurological health, additional studies are needed to determine the role of IS in contributing to adverse neurological health. Pregnant women with kidney disease have an increased risk of delivering preterm, and fetuses born to these women are at an increased risk of being in the neonatal intensive care unit [41]. As women with pregnancies complicated by AKI remain at elevated long-term risk for CKD, targeting uremic toxins after pregnancy may represent a clinically translatable strategy to mitigate disease progression and improve lifelong renal health in this vulnerable population.

## 5. Materials and Methods

All studies were performed using timed-pregnant Sprague-Dawley rats (RRID: MGI:5651135) from Charles River Laboratories (Frederick, MD, USA) due to their well-defined gestational timeline and physiological and placental similarities to humans. Rats arrived on GD10, weighing 220–250 g, and were housed at the Center for Comparative Research at the University of Mississippi Medical Center (UMMC) in a reversed 12:12 light–dark schedule. On the day of arrival, animals were randomly assigned to study groups until each group reached its required numbers. All experiments were in accordance with the National Institute of Health guidelines for the use and care of animals and were approved by the Institutional Animal Care and Use Committee at UMMC under protocol 2022-1198. Only female rats were used in this pregnancy–postpartum study. Animals were excluded from experiments if they were not pregnant.

### 5.1. PR-AKI Model and Treatments

AKI was induced in a subset of rats on GD18 via ischemia–reperfusion, as previously described [15]. To induce AKI, rats were subjected to isoflurane anesthesia and placed in a supine position on a thermo-controlled surgery platform (Braintree Scientific Inc., Braintree, MA, USA). An abdominal midline vertical skin incision was made, and the renal pedicles were isolated to visualize the renal blood vessels. Sterile microvascular clips were placed on the bilateral renal vessels for 45 min to induce ischemia. During this period, saline-soaked gauze pads were placed over the abdomen to prevent the uterine horns and organs in the abdominal cavity from drying out. Rats without AKI served as uncomplicated NP controls, and the abdominal cavity was open for 45 min without renal vessel occlusion. Post-operatively, rats recovered under heat lamps. All rats delivered pups on GD21-22, with pup birth weight and litter size being measured at the time. On PPD2, pups were removed; rats were group housed and transitioned from a pellet-based rat chow diet (0.4% sodium; 0.2% Tryptophan; Teklad 2018) to the ground chow version of Teklad 2018.

A subset of PR-AKI rats was treated with AST-120 (Kremezin^®^, a kind gift from Kureha Corporation, Tokyo, Japan) from PPD10 to PPD80. Rats in the AST-120 group had their rat chow supplemented with AST-120 (5 g/100 g). This dose was chosen based on previously published preclinical studies using AST-120 in virgin female rats [26,27]. Additionally, to assess the role of IS in the absence of ischemia–reperfusion-induced PR-AKI, a group of NP rats received IS (200 mg/kg; Sigma-Aldrich, St. Louis, MO, USA; Catalog Number: I3875) in their drinking water from GD11 until GD19 [33], with a subset receiving AST-120 treatment from PPD10 to PPD80.

At PPM4 (16 weeks postpartum), all rats were euthanized. Kidney weights were recorded and stored for histology (right kidney) or frozen at −80 °C. Serum, plasma, liver, spleen, and the brain were collected and saved at −20 °C or −80 °C for future analysis.

### 5.2. Assessment of Renal Function and Blood Pressure

On GD19, and then monthly beginning in PPM 1, serial measurements assessing body weight and blood pressure were taken until euthanasia at PPM4. After the blood pressure measurement, animals were placed in metabolic cages overnight to collect urine and stool. While in metabolic cages, animals had access to food and water. Urine and stool samples were collected and frozen at −20 °C. To assess blood pressure, rats were placed on a tail warmer plate in restrainers and allowed to acclimate to the testing environment for 15–30 min before blood pressure measurement (CODA noninvasive tail-cuff blood pressure system; RRID: SCR_018585; Kent Scientific, Torrington, CT, USA) [33]. For the blood pressure measurement to be considered valid, at least 60% of the 20 cycles had to be accepted. MAP was the reported outcome for blood pressure. Proteinuria was assessed using the bicinchoninic acid assay (Thermo Fisher, Waltham, MA, USA; Catalog Number: 23250) and reported as mg/day. Serum and urinary creatinine were measured via creatinine assay (Bioassay Systems, Hayward, CA, USA), and serum creatinine was reported as mg/dL. Urinary creatinine was used to measure the protein/creatinine ratio (mg/g). Renal Histopathology: Paraffin-embedded kidney sections were cut into 4 µM sections and stained with Masson’s trichrome to assess renal fibrosis. The percentage of blue staining in the renal cortex was measured in trichrome sections (10 images per section) to evaluate renal fibrosis, as previously described [15]. All images were digitally captured with, an EVOS XL microscope (Invitrogen, Thermo Fisher, Waltham, MA, USA, and analyzed ImageJ version 1.54p, and scored by blind assessors. The percent of blue (amount of fibrosis) was reported.

Uremic Toxin Measurement via LC-MS and ELISA: LC-MS analysis of urinary IS levels was performed by the Mississippi INBRE metabolomics and lipidomics core using LC/MS- or HPLC-grade supplies and optimized protocols (see Appendix A for details). Briefly, urine samples were diluted 1:10 in LC/MS grade H_2_O before processing to minimize matrix effects. Samples were diluted 1:5 with an internal standard solution containing d4-IS, vortexed for 2 min, and centrifuged at 10,000× *g* for 10 min at RT. Supernatants were diluted 1:5 in 0.1% formic acid (in water). Different concentrations of calibration standards in 50% methanol were prepared for IS, then combined and diluted 1:100 in 15% acetonitrile to final concentrations of 1, 3, 10, 30, 100, 300, 1000, and 3000 ng/mL (for IS) and spiked with d4-IS to a final concentration of 100 ng/mL. The samples were analyzed in triplicate on a TSQ Quantis Plus triple-quadrupole MS (Thermo Fisher Scientific, San Jose, CA, USA) equipped with the Acquity UPLC system (RRID: SCR_027039; Waters, Milford, MA, USA). Liquid chromatography separation was carried out using an Acquity BEH C18 column (2.1 × 100 mm, 1.7 µm) fitted with an Acquity BEH C18 VanGuard pre-column (2.1 × 5 mm, 1.7 µm) (Waters, Milford, MA, USA) at 40 °C using a column oven and with an injection volume of 2 µL. The mobile phases consisted of 10 mM ammonium formate (pH 4.3) (A), and acetonitrile (B). An isocratic elution was employed with mobile phase A at 85% and mobile phase B at 15%. The total run time and flow rate were 6 min and 0.3 mL/min, respectively. The column eluate was directed into the MS using an electrospray ionization interface in negative mode. Xcalibur software (Thermo Fisher Scientific, San Jose, CA, USA) was utilized for data acquisition and processing. LC-MS data were normalized to urine output and is represented as nM of toxin/mL/day. Total circulating IS was measured according to the manufacturer’s guidelines using an ELISA (MyBioSource; San Diego, CA, USA; Catalog Number: MBS3809027).

### 5.3. Statistical Analysis

Comparisons between groups and/or study time points were analyzed using one-way ANOVA, two-way ANOVA, or mixed models to evaluate the relationship between experimental groups and/or study time points. All data were normally distributed as assessed by the Shapiro–Wilk test and all groups had equal variances as assessed by Bartlett’s test. Post hoc analysis was conducted with Tukey’s multiple comparison test or Student’s *t*-test. A mixed model analysis was performed due to premature deaths among some animals. *p* < 0.05 was considered statistically significant. Results are presented as mean ± standard error mean, and statistical analysis and figure generation were conducted using GraphPad Prism 10 (RRID: SCR_002798). An a priori sample size calculation was conducted. Our Cohen’s d was based on prior data published by our group, and using a power = 80%, α = 0.05, the sample size was 4–9, depending on the outcome.

## Figures and Tables

**Table 1:** Blood pressure and renal function parameter measurements.

	NP	PR-AKI	PR-AKI+AST-120
**GD19**			
MAP (mmHg)	94.6 ± 2.86	99.4 ± 2.56	
Urine Output (mL/day)	24.2 ± 1.50	14.9 ± 1.41 ^a^	
Proteinuria (mg/day)	4.2 ± 0.52	14.6 ± 2.07 ^a^	
Protein/Creatinine Ratio (mg/g)	13.9 ± 1.54	48.1 ± 7.92 ^a^	
**PPM2**			
MAP (mmHg)	107.8 ± 4.90	118.3 ± 2.83	103.9 ± 3.6 ^b^
Urine Output (mL/day)	18.0 ± 1.41	12.4 ± 1.53	15.8 ± 1.78
Proteinuria (mg/day)	6.0 ± 1.06	29.1 ± 6.19 ^a^	16.0 ± 2.98
Protein/Creatinine Ratio (mg/g)	11.9 ± 3.46	43.5 ± 7.23 ^a^	19.3 ± 3.15 ^b^
**PPM4**			
MAP (mmHg)	104.2 ± 2.78	119.9 ± 5.49 ^a^	113.9 ± 5.53
Urine Output (mL/day)	21.2 ± 2.29	13.7 ± 1.59 ^a^	21.4 ± 2.59
Proteinuria (mg/day)	6.1 ± 1.01	16.8 ± 1.77 ^a^	13.7 ± 2.68
Protein/Creatinine Ratio (mg/g)	10.1 ± 3.17	23.7 ± 11.40	20.5 ± 2.74
Serum Creatinine (mg/dL)	0.64 ± 0.05	1.03 ± 0.13 ^a^	0.54 ± 0.12 ^b^

^a^ NP vs. PR-AKI, ^b^ PR-AKI vs. PR-AKI+AST-120.

**Table 2:** Blood pressure and renal function parameter measurements among NP+IS rats.

	NP	NP+IS	NP+IS+AST-120
**GD19**			
MAP (mm/Hg)	94.6 ± 2.86	96.5 ± 2.77	
Urine Output (mL/day)	24.2 ± 1.50	14.8 ± 1.42 ^a^	
Proteinuria (mg/day)	4.2 ± 0.52	19.1 ± 2.76 ^a^	
Protein/Creatinine Ratio (mg/g)	13.9 ± 1.54	15.9 ± 16	
**PPM2**			
MAP (mm/Hg)	107.8 ± 4.90	113.5 ± 4.13	100.3 ± 2.42 ^b^
Urine Output (mL/day)	18.0 ± 1.41	11.5 ± 1.87	12.1 ± 0.93
Proteinuria (mg/day)	6.0 ± 1.06	20.9 ± 4.14 ^a^	17.8 ± 2.06 ^c^
Protein/Creatinine Ratio (mg/g)	11.9 ± 3.46	15.9 ± 3.98	11.2 ± 1.19
**PPM4**			
MAP (mm/Hg)	104.2 ± 2.78	113.8 ± 5.60	96.0 ± 5.64 ^b^
Urine Output (mL/day)	21.2 ± 2.29	15.5 ± 1.89 ^a^	14.8 ± 2.22
Proteinuria (mg/day)	6.1 ± 1.01	17.6 ± 2.36 ^a^	9.7 ± 1.92 ^b^
Protein/Creatinine Ratio (mg/g)	10.1 ± 3.17	16.2 ± 4.94	11.7 ± 1.60
Serum creatinine (mg/dL)	0.64 ± 0.05	0.94 ± 0.09	0.57 ± 0.08

^a^ NP vs. NP+IS, ^b^ NP+IS vs. NP+IS+AST-120, ^c^ NP vs. NP+IS+AST-120.

## Data Availability

The original contributions presented in this study are included in the article. Further inquiries can be directed to the corresponding author.

## Abbreviations

The following abbreviations are used in this manuscript:

CKD	Chronic kidney disease
GD	Gestational day
IS	Indoxyl sulfate
MAP	Mean arterial pressure
NP	Normal pregnant
OAT	Organic anion transporter
pCS	p-cresol sulfate
PPD	Postpartum day
PPM	Postpartum month
PR-AKI	Pregnancy-related acute kidney injury

## Appendix A

### Appendix A.1. Experimental Details of LC-MS (Sample Processing)

Rat urine samples were diluted 1:10 with LC/MS grade H_2_O before processing to minimize matrix effects. Samples were then diluted 1:5 with an internal standard solution containing d_4_-indoxyl sulfate (625 ng/mL in LC/MS grade acetonitrile), vortexed for 2 min, and centrifuged at 10,000× *g* for 10 min at room temperature (RT). The resulting supernatants were diluted 1:5 with 0.1% LC/MS grade formic acid (in LC/MS grade H_2_O), ensuring compatibility with the mobile phase composition (85% aqueous: 15% organic).

### Appendix A.2. Experimental Details of LC-MS (Preparation of Metabolite Stock Solutions and Calibration Standards)

Stock solutions of IS, p-CS, d_4_-IS, d_7_-p-CS were prepared in 50% HPLC-grade methanol. Intermediate solutions of these metabolites were also prepared in 50% HPLC-grade methanol to be used to prepare the internal standard solution for sample processing and calibration standards Starting calibration standards were prepared at concentrations of 0.1, 0.3, 1, 3, 10, 30, 100, and 300 μg/mL (for IS) and 0.03, 0.1, 0.3, 1, 3, 10, 30, and 100 μg/mL (for p-CS) in 50% HPLC-grade methanol and then diluted 1:100 together in 15% LC/MS grade acetonitrile to the final concentrations of 1,3,10,30,100,300,1000, and 3000 ng/mL (for IS) and 0.3, 1, 3, 10, 30, 100, 300, 1000 ng/mL (for p-CS). d_4_-IS and d_7_-p-CS were added to each standard to a final concentration of 100 ng/mL. Compatibility with the mobile phase composition was ensured.

### Appendix A.3. Experimental Details of LC-MS Analysis

The metabolite samples were analyzed in triplicate on a TSQ Quantis Plus triple-quadrupole mass spectrometer (Thermo Fisher Scientific, San Jose, CA, USA) equipped with an Acquity UPLC system (Waters, Milford, MA, USA). Chromatographic separation was carried out using an Acquity BEH C18 column (2.1 × 100 mm, 1.7 µm) fitted with an Acquity BEH C18 VanGuard pre-column (2.1 × 5 mm, 1.7 µm) (Waters, Milford, MA, USA) at 40 °C using a column oven and with an injection volume of 2 µL. The mobile phases consisted of 10 mM ammonium formate acidified with LC/MS grade formic acid to a pH of 4.3 (in LC/MS grade H_2_O) (A) and LC/MS grade acetonitrile (B). An isocratic elution was employed with mobile phase A at 85% and mobile phase B at 15%. The total run time and flow rate were 6 min and 0.3 mL/min, respectively. The column eluate was directed into the mass spectrometer using an electrospray ionization interface in negative mode. The MS conditions were set as follows: spray voltage = 2000 V, vaporizer temperature = 300 °C, sheath gas pressure = 50 (arbitrary units), auxiliary gas pressure = 10 (arbitrary units), capillary temperature = 300 °C, collision gas pressure = 1.5 mTorr, scan time = 0.1 s and full width at half maximum (FWHM) of 0.7 *m*/*z* and 1.2 *m*/*z* for the first and third quadrupole, respectively. Samples were run in selected reaction monitoring (SRM) mode using precursor-to-product ion transitions: *m*/*z* 212.0 → 80.4 for IS (collision energy = 27 V), *m*/*z* 215.9 → 80.4 for d_4_-IS (collision energy = 31 V), *m*/*z* 186.9 → 107.5 for p-CS (collision energy = 26 V), and *m*/*z* 193.9 → 114.6 for d_7_-p-CS (collision energy = 24 V). Xcalibur software (version 4.5.474.0) (Thermo Fisher Scientific, San Jose, CA, USA) was utilized for data acquisition and processing.
